# A rank weighted classification for plasma proteomic profiles based on case-based reasoning

**DOI:** 10.1186/s12911-018-0610-1

**Published:** 2018-05-31

**Authors:** Amy M. Kwon

**Affiliations:** Big Data Science, Division of Economics & Statistics, College of Public Policy, Korea University, Sejong, Korea

**Keywords:** Case-based reasoning, Plasma proteomic profiles, Classification, Rank

## Abstract

**Background:**

It is a challenge to precisely classify plasma proteomic profiles into their clinical status based solely on their patterns even though distinct patterns of plasma proteomic profiles are regarded as potential to be a biomarker because the profiles have large within-subject variances.

**Methods:**

The present study proposes a rank-based weighted CBR classifier (RWCBR). We hypothesized that a CBR classifier is advantageous when individual patterns are specific and do not follow the general patterns like proteomic profiles, and robust feature weights can enhance the performance of the CBR classifier. To validate RWCBR, we conducted numerical experiments, which predict the clinical status of the 70 subjects using plasma proteomic profiles by comparing the performances to previous approaches.

**Results:**

According to the numerical experiment, SVM maintained the highest minimum values of Precision and Recall, but RWCBR showed highest average value in all information indices, and it maintained the smallest standard deviation in F-1 score and G-measure.

**Conclusions:**

RWCBR approach showed potential as a robust classifier in predicting the clinical status of the subjects for plasma proteomic profiles.

## Background

Case-based reasoning (CBR) is an artificial intelligent approach based on an inference technique that is said to be the most effective method to construct an expert system [1]. When a target case occurs, CBR is mainly performed according to the following four procedures: retrieving, reusing, revising and retaining [2, 3]. It solves a target problem by revising the solution with the previous cases in similar situations retrieved from the case-base, and the target case is retained in the case-base for the next problem once the problem is solved. Thus, up-to-date case-base is always maintained in CBR system. The CBR system has been applied in many learning or problem-solving techniques of real-world applications. In particular, the prediction techniques based on CBR can be more appropriate in bio-medical field than other fields because CBR has less risk of overfitting in prediction, and medical cases can’t be often explained by general patterns of the case-base. It is important to classify the plasma proteomic profiles solely depending on their shapes because their distinct patterns of profiles are regarded as a potential biomarker according to clinical status [4]. However, plasma proteomic profiles may be a typical example not following the general patterns which lead to poor accuracies in prediction by classification methods based on overall means of similarity due to large within-subject variance, and there is no gold standard to analyze the plasma proteomic profiles yet. The present study conducts a CBR based classification method with the plasma proteomic profiles which does not make decision for classification depending on the overall mean. However, CBR often also shows lower prediction performance compared to other learning techniques. Previous studies proposed some methods to improve the performance of CBR. Those studies were primarily focused on either weight optimization methods [5–9] or feature (or subset) selection methods [10, 11], and one study proposed a hybrid generic approach to optimize the both with the number of neighbor cases to compute in the case retrieval procedure of CBR [12]. The meaningful set of features is often predetermined by experts in bio-medical fields, and the most similar case may result in the best accuracy in prediction when output values of each feature are wide-spread like plasma proteomic profiles. If that is the case, a proper weight optimization may only enhance the prediction performance of CBR. The weights are optimized either subjectively or objectively. Subject weights are typically allocated according to the preference scores or information of experts such as Delphi method [5]. Objective weights can be allocated by entropy method [7], statistical method [8] or they can be optimized while proceeding algorithms such as generic algorithm (GA) [12] or neuralnet (NN) [6]. Among these approaches, NN needs a large number of inter-connected neurons to allocate weights, so small or moderate-size samples may not attain a standard structure of NN [9]. GA is also criticized due to premature convergence or low reliability [9]. A weight by a statistical method was allocated by the proportion of Wald’s statistics [8] which is obtained by assuming asymptotically normal distributions of parameters. The present study proposes a non-parametric weight allocation method without using normality assumption. We investigate the accuracy of a CBR based classification with plasma proteomic profiles to diagnose cervical cancer, and observe the enhancement of the prediction performance of the CBR classifier by allocating feature weights. To validate our approach, we also conduct previous weight allocation methods for the CBR classifier together with the plasma proteomic profiles. The paper is organized as follows. After introduction, section 2 briefly describes the CBR system and reviews previous studies. Section 3 presents the proposed method using Rank, and section 4 describes our data schemes and empirical results. At last, the conclusion and further research are discussed in section 5.

## Methods

### The CBR classifier with plasma proteomic profiles

**Fig. 1:**
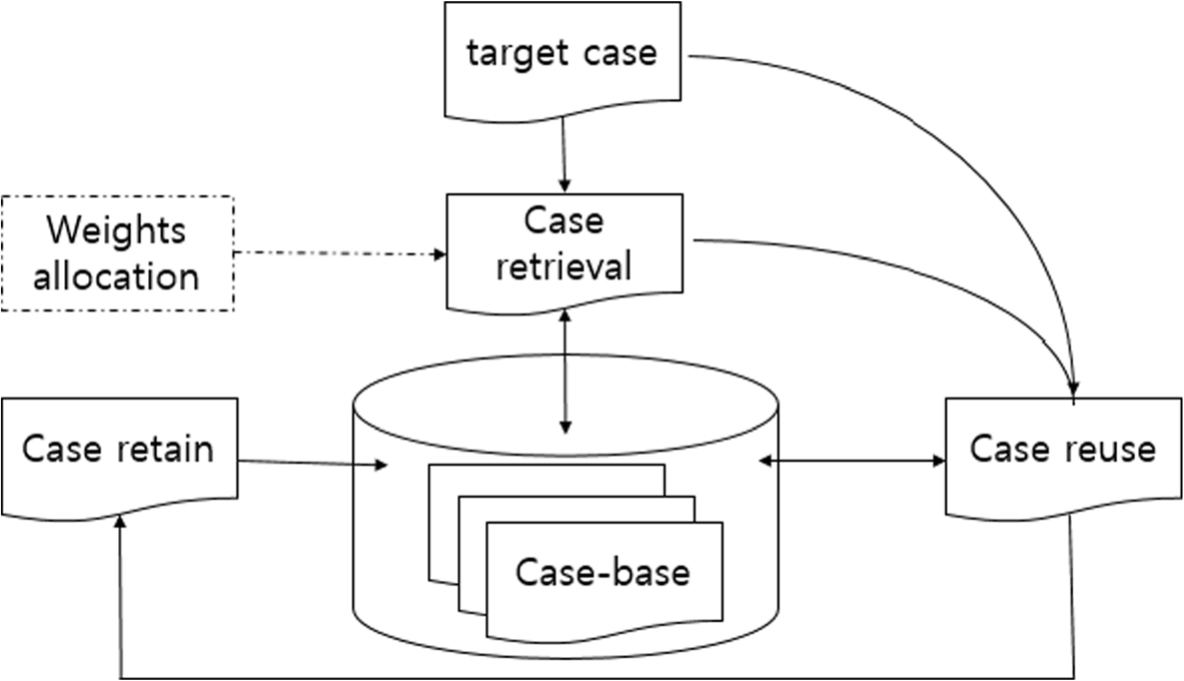
Problem solving process of CBR Classifier. The CBR classifier finds the solution of the problem by retrieving similar cases stored in the case-base to the target problem as described

The general problem-solving process by the CBR classifier is described in Fig. 1. The CBR classifier describes a target problem using old experiences, and finds a solution of the problem by retrieving similar cases stored in the case-base to the target problem where the case-base is the specific knowledge base of past experiences. The case is typically retrieved by learning techniques for the CBR classifier, and the most common technique is *k*-nearest neighbor (NN). The original CBR classifier uses 1-NN which retrieves the most similar case from the case-base to the target problem. The problem is adapted from the retrieved cases, and is revised. Once the problem is solved, the cases are retained. The CBR classifier with plasma proteomic profiles maintained the same scheme. The problem is to identify the class of a target case by comparing the pattern of the target case with those in the case-base where the case-base consists of trained samples with their class-labels. The case is retrieved from the case-base by *k*-nearest neighbor (NN) to solve the problem, and the target case as well as the retrieved case are stored in the case-base once the class of the target case is determined.

### Prior studies for weight optimization

The original classifier assesses the similarity of a target case with cases in the case-base under the assumption that all features are equally likely important. However, it may be practical to think of the relative importance among the features, so some researches differently allocated the weights on features considering the relative importance. Since different weights for the attributes can vary the distribution of the overall similarities among the cases, the retrieved cases by the CBR classifier can be different depending on the altered distribution of the similarities. Regarding that matter, the weight allocation or optimization is closely related with the performance of the CBR classifier. In particular, the weight allocation or optimization techniques have gained attention as a way to enhance the performance of the CBR classifier in previous studies.

DELPHI method is one of the most common approaches to allocate feature weights to the CBR classifier. DELPHI method directly reflects experts’ opinions about the features as the corresponding weights like Gu et al. [13] or Chang et al. [5], so the weights can be changed by the point of view of the subjects. Alternatively, weights have been objectively allocated using information gain or entropy. Cardie and Howe [14] first selected a set of relevant features using a decision tree, and assigned the weights with information gain to the feature which was chosen by the tree. Ahn and Kim [12] encoded feature weights with numbers from 0 to 7 which represented the relative importance of the features. These numbers were processed as 3-bit binary numbers and transformed into floating decimal numbers (x_f_) for weights. Zhao et al. [7] used information entropy for feature weights to select suppliers. They computed the average regression coefficients to seek the integrated average index of each supplier, and calculated both the information gain in ID3 of the decision tree and the entropy. These values were later standardized as the numbers in the range of [0,1] for weights. Besides, Liang et al. [8] optimized feature weights by a statistical approach. They fitted features with binary logistic regression, and computed the Wald statistics of parameter estimates for the features. Then, the statistics are standardized by dividing them by the sum of all the statistics before they are allocated to the features as the weights. Suitable weights may vary depending upon problems we encountered. Prior studies about the weight optimization or allocation methods are summarized in Table 1.

**Table 1:** Prior studies about the weight optimization methods

Authors	Year	Methods	Weights
Cardie & Howe [14]	(1997)	Information gain	G(f) ^a^
Ahn & Kim [12]	(2009)	Relative importance [0-7]	$\frac{x_f}{\sum \limits_{f=1}^m{x}_f}$
Gu et al. [13]	(2010)	Delphi method	–
Chang et al. [5]	(2011)	Delphi method	–
Zhao et al. [7]	(2011)	Entropy method	$\frac{entropy_f}{\sum \limits_{f=1}^m{entropy}_f}$
Liang et al. [8]	(2012)	Logistic regression	$\frac{Wald_f}{\sum \limits_{f=1}^m{Wald}_f}$

^a^ indicates information gain of the f-th feature, and entropy is defined as $-\sum \limits_i{p}_i\bullet {\log}_2{p}_i$

### Rank-based weight optimization

#### Distance functions and problem setting

A typical similarity or dissimilarity measure is a distance metric, and it is crucial to learn a good distance metric to represent the similarity or dissimilarity in feature space although there are considerable researches on distance metrics [15–17]. Some researches have been focused on the comparison of their impacts on the performance in classification with known public database [18, 19]. However, no single similarity or dissimilarity showed dominantly superior to the others in all methods in their studies [18, 19]. Most classifiers try to use a distance metric that keeps data points close if the class labels are the same while keeps distance from the data points if the class labels are different. The goal of the CBR classifier is to predict a class label of a target case of ${\overrightarrow{x}}_0$ by retrieving the most similar case from the case-base using the proper distance metric. Let $\chi =\left\{{\overrightarrow{x}}_1,\cdots, {\overrightarrow{x}}_n\right\}$ be a collection of n data points in the case-base with the known class labels of *C* = {*c*_1_, ⋯, *c*_*n*_} where ${\overrightarrow{x}}_i\in {R}^m$ and *c*_*i*_ ∈ {1, ⋯, *K*}. The CBR classifier typically adapts the *k*-NN approach to retrieve the similar cases to the target case with a given *k*. The *k*-NN approach assumes that the class conditional probability in the nearest neighbors to ${\overrightarrow{x}}_0,N\left({\overrightarrow{x}}_0\right)$, is constant, and tries to maintain consistency in predicting class labels for ${\overrightarrow{x}}_0$ by obtaining its neighborhood as follows where I(·) is an indicator function.

1 $$ p\left(j\left|{\overrightarrow{x}}_0\right.\right)=\frac{\sum_{i=1}^nI\left({\overrightarrow{x}}_i\in N\left({\overrightarrow{x}}_0\right)\right)I\left({c}_i=j\right)}{\sum_{i=1}^nI\left({\overrightarrow{x}}_i\in N\left({\overrightarrow{x}}_0\right)\right)} $$

The global distance between the target case and any case in the case-base is computed by summing up the local distances to determine the nearest neighbors for the target case on Eq. (1). The local distance is computed for each feature between the target case and any case in the case-base by the pre-defined local distance metric, and the types of local distance metric do not have to be the same among the features. Euclidean distance metric is typically used to compute the physical distance between the two data points, but it suffers in the case that vectors of data points aren’t linearly distributed like default measurements of proteomic profiles. On the contrary, Fre’chet distance metric is known to be useful to measure the distance between the data points when the vectors of those data points lie on the non-linear curve [20]. According to the characteristics of the feature types, the distance metric consists of either Euclidean distance metric or Fre’chet distance metric for the present study. Euclidean distance metric and Fre’chet distance metric are defined for the feature, f, as follows where ${\overrightarrow{x}}_i^{(f)}$ and ${\overrightarrow{x}}_0^{(f)}$ are sub-vectors of any data point in the case-base and the target case consisting of the feature, f, respectively.

2 $$ {d}_f\left({\overrightarrow{x}}_i^{(f)},{\overrightarrow{x}}_0^{(f)}\right)={\left({\overrightarrow{x}}_i^{(f)}-{\overrightarrow{x}}_0^{(f)}\right)}^T\left({\overrightarrow{x}}_i^{(f)}-{\overrightarrow{x}}_0^{(f)}\right) $$

3 $$ {d}_f\left({\overrightarrow{x}}_i^{(f)},{\overrightarrow{x}}_0^{(f)}\right)=\kern0.5em \underset{\alpha, \beta }{\operatorname{inf}}\kern0.5em \underset{t^{\prime}\in \left[0,1\right]}{\max}\kern0.5em d\left({\overrightarrow{x}}_i^{(f)}\left(\alpha \left({t}^{\prime}\right)\right),{\overrightarrow{x}}_0^{(f)}\left(\beta \left({t}^{\prime}\right)\right)\right) $$

#### Conversion to rank-order information

Plasma proteomic profiles have the large within-subject variance. Although the class labels are the same, the profiles can be distributed over a considerable extent as well as they are not following the general pattern. We determined the proximity of the cases using the global similarity based on rank-order information of the distances [21] instead of using the distance itself to enhance robustness in predicting the class label of the target case in the present study. The similarity is computed as follows where N′ is the number of cases having a unique ranking-order in the case-base and ω_*f*_ is an unknown weight for a feature of *f*.

4 $$ S\left({\overrightarrow{x}}_i,{\overrightarrow{x}}_0\right)=\sum \limits_{f=1}^m\omega f\cdot \left[\frac{N^{\prime }-\operatorname{rank}\left({d}_f\left({\overrightarrow{x}}_i^f,{\overrightarrow{x}}_0^f\right)\right)}{N^{\prime }-1}\right] $$

According to Eq. (4), the higher the rank, the greater the similarity between the *i*-th case and the target case.

#### Weight optimization

Every feature is equally likely important to the original CBR classifier. Since the original CBR classifier often showed lower predictability, there have been some researches to improve the predictability by assigning different weights to the features according to their relative importance. In the same line of thoughts, we adopted the different weights to the feature in calculating the similarity, and optimized the weights according to the objective function from Wilcoxon’s rank sum test statistics. The ability of the objective function is mainly influenced by the feature weights, and the weights are determined to maximize the ability of the objective function to differentiate the cases having different class labels. Wilcoxon’s rank sum test is a non-parametric test to assess the difference of the mean ranks for two samples, and it is known to be useful when outliers exist in the observations compared to the parametric tests. The similarity is regarded as a function of ranks in the present study because the similarity is computed according to the corresponding rank-order information of the distances for features between the target case and any case in the case-base. Thus, the weights can be naturally allocated to the features in the similarity measure maintaining the same property from the objective function based on Wilcoxon’s rank sum statistics. The objective function for the present study can be summarized as follows where n_1_ is the number of cases having the class label of 1 when the class labels are denoted as either 0 or 1 and the number of classes, J, is set to 2.

5 $$ {\displaystyle \begin{array}{l}\arg \kern0.5em {\max}_{\omega_f:f=1,\cdots, m}\kern4em {\sum}_{f=1}^m{\omega}_f\cdot {r}_f\\ {}\mathrm{where}\kern12em {r}_f={\sum}_{i=1}^{n_1}\operatorname{rank}\left({d}_f\left({\overrightarrow{x}}_i^{(f)},{\overrightarrow{x}}_0^{(0)}\right)\right)-\frac{n_1\cdot \left({n}_1+1\right)}{2}\\ {}\mathrm{constraint}\kern0.5em \mathrm{to}\kern6.5em \Big\{\begin{array}{c}0\le {\omega}_f\le 1\\ {}{\sum}_{f=1}^m{\omega}_f=1\end{array}\operatorname{}\end{array}} $$

On Eq. (5), as the probability increases that the two groups of the cases are truly drawn from the population-cases having the different class labels, the corresponding feature weight of ω_f_ becomes large because the resulted statistics, *W*, is large. The significance of the test statistics is directly represented by the magnitude of the corresponding *p*-value, so the feature weights can be computed using the magnitudes of *p*-values from the test statistics as follows.

6 $$ {\omega}_f=\frac{1-p\left(|W|\ge {r}_f\right)}{\sum_{f=1}^m1-p\left(|W|\ge {r}_f\right)} $$

where *W* denotes the test statistics of the Wilcoxon’s rank sum test. The feature weights from Eq. (6) are used to compute the similarity of Eq. (4).

#### Application and experiments

##### Data description

The proteomic profiles were obtained from the blood plasma samples which were collected from recruited subjects at the University of Louisville, KY, USA. Total 70 female subjects were recruited for this study, and 50% of those subjects were diagnosed with cervical carcinoma while the others are healthy controls, without any known diseases. The study protocol was approved by the institutional review board of the University of Louisville, and informed consent forms were voluntarily signed by the participants. The origin of the data can be referred to [22], and the secondary data was used for the study. The default output measurement of the proteomic profiles was the excess heat capacity (ΔC_p_), which were recorded at the different temperatures from 45 to 90 °C by incrementally adding 1 °C to the previous measuring temperature. The proteomic profiles were preprocessed prior to the analysis. The excess heat capacity (ΔC_p_) as the default measurement is a vector of real numbers of length 451 and it typically shows one or two peaks on the range of temperatures during the experiment. We newly extracted 5 features from the pre-processed data besides the excess heat capacity. The feature information is summarized in Table 2. The class information for each proteomic profile was labeled as either ‘control’ or ‘cancer’ according to the clinical status of the corresponding subject. On Table 2, PEAK1 and PEAK2 indicate those peaks, and T1 and T2 are temperatures that those peaks occur at where {PEAK1, PEAK2, T1, T2} were estimated by Gaussian kernel regression from the excess heat capacity patterns. IND indicates a set of 451 individual measurement of the excess heat capacity, and IR is a binary value indicating the initial directional tendency of the excess heat capacity as the temperature increases. IR is 1 if the directional tendency is positive, 0 otherwise.

**Table 2:** Description of the features

Features (Abbreviation)	Type	Contents
Initial Response (IR)	Binary number	0: decreasing
		1: increasing
Temperature 1 (T1)	Real number	Range [45 - 55]
Temperature 2 (T2)	Real number	Range [56 - 90]
Maximum Peak at T1 (PEAK1)	Real number	Range [0 - ∞]
Maximum Peak at T2 (PEAK2)	Real number	Range [0 - ∞]
A set of individual *∆C*_*p*_ (IND)	A vector of real numbers	Range [0 - ∞]

##### Numerical experiments

The purpose of the numerical experiments is to study the performance of the CBR classifier in prediction with the plasma proteomic profiles by comparing with the previous approaches. In particular, we observed whether the rank-based feature weights enhance the performance of the CBR classifier with proteomic profiles or not. As reference methods, two common machine learning methods, *k*-NN (*k* -NN) and support vector machine (SVM), a statistical approach using the composite coefficient [23], and three CBR approaches weighted by different allocation methods in previous studies [7, 8] were conducted to validate the performance of the proposed CBR approach for the present study. The number of neighbors for *k* -NN was 5 which was determined by cross-validation (CV) with training samples, and SVM was conducted based on the radial basis kernel.

The statistical model was introduced to show the difference of the plasma proteomic profiles between two groups having different clinical status using a composite coefficient which was a weighted product of an average probability being in the same group of the reference sample and Pearson’s correlation coefficient. In the present study, this model was conducted with the default setting of the composite coefficient as in the literature [23]. Namely, the reference set was composed with the cases in the ‘control’ class for this model, and the weight factor of the composite coefficient was set to 1 as described in the literature. This classification model is abbreviated as (SCUCC) indicating statistically classified using the composite coefficient for the experiments as a reference method. Among the CBR approaches, the first model is a classical CBR approach (CLCBR), which gives attributes equal-weights and uses *1*-NN for the case retrieval. This model would be the base model to examine the effect of the CBR classifiers having different weights on features. ETCBR and LWCBR are the weighted CBR approaches. The feature weights were allocated with standardized entropy value in ETCBR [7]. In the present study, IR is Berno’ulli and the other features are assumed as normal distribution. The computed entropy was standardized by dividing each entropy by the sum of all entropy values prior to allocation, and 1-NN was used for the case retrieval. LWCBR indicates a weighted CBR approach from logistic regression model. This model adopted standardized Wald statistics of the regression coefficients for feature weights by fitting the observation with binary logistic regression. Namely, the Wald statistics were divided by the sum of the all statistics before they were allocated to the features [8], and also used 1-NN for the case retrieval. Logistic regression is a typical parametric approach and Wald statistics are derived from the regression coefficients under the asymptotic normal assumption, so this model can be a good reference to observe the performance of the proposed feature weights. The proposed CBR approach is abbreviated as RWCBR indicating a rank-weighted CBR approach. As described in the above sections, the feature weights were computed from Wilcoxon’s rank sum test and the most similar case was retrieved as the other CBR approaches.

The data set of proteomic profiles consists of the pre-defined features on Table 2 with 70 subjects, and the class labels are fully given with the number of classes as two. The data set was randomly partitioned into five equal-sized subsets for 5-fold CV. At each fold, a subset was selected as a test set, and the other four subsets became a training set where the proportion of the cases were equally distributed from the two classes during the experiments. The feature weights for the ETCBR, LWCBR and RWCBR were estimated with the training set, and the optimized weights were allocated to the features in the test set.

## Results

Each fold has the same size of cases in the test set and the training set as 14 and 56, respectively during the experiments. The estimated feature weights by ETCBR, LWCBR and RWCBR at each fold are summarized in Table 3. The performances of the seven different models were evaluated at each fold in terms of Precision, Recall, F-1 score, [24, 25] and G-measure [25]. The estimated weights in Table 3 were allocated to the features when the CBR classifiers retrieved the most similar case from the case-base by ETCBR, LWCBR and RWCBR. The information indices of Precision, Recall, F-1 score and G-measure are defined as follows.

$$ {\displaystyle \begin{array}{c}\mathrm{Precesion}=\frac{\sum \mathrm{true}\kern0.5em \mathrm{positive}}{\sum \left(\mathrm{true}\kern0.5em \mathrm{positive}+\mathrm{false}\kern0.5em \mathrm{positive}\right)}\\ {}\mathrm{Recall}=\frac{\sum \mathrm{true}\kern0.5em \mathrm{positive}}{\sum \left(\mathrm{true}\kern0.5em \mathrm{positive}+\mathrm{false}\kern0.5em \mathrm{negative}\right)}\\ {}\mathrm{F}\hbox{-} 1\kern0.5em \mathrm{score}=2\cdot \frac{\mathrm{precision}\cdot \mathrm{recall}}{\left(\mathrm{precision}+\mathrm{recall}\right)}\\ {}\mathrm{G}\hbox{-} \mathrm{measure}=\sqrt{\mathrm{precision}\cdot \mathrm{recall}}\end{array}} $$

**Table 3:** Estimated feature weights

Fold	Model	IR	PEAK1	PEAK2	T1	T2	IND
I	ETCBR	0.0067	0.0145	0.0347	0.0595	0.0220	0.8625
	LWCBR	0.0158	0.1145	0.3845	0.0960	0.0179	0.3713
	RWCBR	0.1704	0.1890	0.1488	0.1886	0.1596	0.1436
II	ETCBR	0.0089	0.0311	0.0477	0.0766	0.0314	0.8042
	LWCBR	0.0877	0.0752	0.2550	0.0016	0.3244	0.2561
	RWCBR	0.2190	0.2222	0.0954	0.2203	0.1325	0.1106
III	ETCBR	0.0068	0.0265	0.0391	0.0654	0.0243	0.8377
	LWCBR	0.0175	0.1780	0.2481	0.1188	0.1584	0.2793
	RWCBR	0.1274	0.2603	0.1144	0.2837	0.1092	0.1051
IV	ETCBR	0.0076	0.0259	0.0383	0.0643	0.0247	0.8392
	LWCBR	0.0001	0.3524	0.0682	0.4252	0.1484	0.0056
	RWCBR	0.1627	0.2266	0.1279	0.2206	0.1233	0.1388
V	ETCBR	0.0065	0.0223	0.0345	0.0581	0.0213	0.8573
	LWCBR	0.0250	0.0036	0.1628	0.2751	0.3219	0.2116
	RWCBR	0.2205	0.2186	0.1024	0.2334	0.1076	0.1175

With the retrieved case for each target case, the class label of the target case was predicted according to Eq. (1), and the prediction results were used to compute the information indices at each fold according to the above definitions.

The resulting information indices at each fold are summarized on Table 4, and the comprehensive statistics using the minimum (MIN), average, (AVG), standard deviation (STD) and the maximum (MAX) for each index are summarized on Table 5. Among the models, RWCBR and SVM consistently showed good performances over different sample sets in predicting the class labels with plasma proteomic profiles in comparison with others, but the performance of RWCBR was slightly better. In case of Precision and Recall indices, LWCBR had the biggest range from the minimum of 33% to the maximum of 80% and from the minimum of 29% to the maximum of 100%, respectively while SVM had the smallest range from the minimum of 85.7% to the maximum of 100% in both indices. However, RWCBR showed the highest average value of 91% among the all models in both Precision and Recall, and the performance was maintained at least 71%. ETCBR and RWCBR showed better performance than CLCBR in both Precision and Recall, but LWCBR worked poor in comparison with CLCBR by showing lower mean values in all indices although we generally expected a weighted CBR approach to perform better than CLCBR. Comparing CLCBR to SCUCC, a statistical approach, average precision index of CLCBC was lower, but average recall index was higher. F-1 score and G-measure were similar between the two, so it appears that CBR approaches do not always work well with plasma proteomic profiles. Regarding F1-score and G-measure, SVM also maintained the shortest ranges, but RWCBR showed the best performance at most aspects of summary statistics among the seven models. In particular, it maintained the smallest standard deviations in comparison with the other models.

**Table 4:** Information indices by 5-Fold CV

Fold	Measures	K-NN	SVM	SCUCC	CLCBR	ETCBR	LWCBR	RWCBR
I	Precision	0.5000	0.8571	1.0000	0.6667	1.0000	0.8000	1.0000
	Recall	0.7143	0.8571	0.2857	0.5714	0.7143	0.5714	0.7143
	F1-score	0.5882	0.8571	0.4444	0.6154	0.8333	0.6667	0.8333
	G-measure	0.5976	0.8571	0.5345	0.6171	0.8452	0.6761	0.8452
II	Precision	0.8571	0.8571	1.0000	0.8000	0.7500	0.3333	1.0000
	Recall	0.8571	0.8571	0.8571	0.5714	0.8571	0.2857	0.8571
	F1-score	0.8571	0.8571	0.9231	0.6667	0.8000	0.3077	0.9231
	G-measure	0.8571	0.8571	0.9258	0.6761	0.8018	0.3086	0.9258
III	Precision	0.6000	0.8571	0.8333	0.7500	0.8333	0.7778	0.7778
	Recall	0.8571	0.8571	0.7143	0.8571	0.7143	1.0000	1.0000
	F1-score	0.7059	0.8571	0.7692	0.8000	0.7692	0.8750	0.8750
	G-measure	0.7171	0.8571	0.7715	0.8018	0.7715	0.8819	0.8819
IV	Precision	0.7500	1.0000	1.0000	1.0000	0.8750	0.7778	1.0000
	Recall	0.8571	1.0000	0.5714	1.0000	1.0000	1.0000	1.0000
	F1-score	0.8000	1.0000	0.7273	1.0000	0.9333	0.8750	1.0000
	G-measure	0.8018	1.0000	0.7559	1.0000	0.9354	0.8819	1.0000
V	Precision	0.5455	0.8571	0.7000	0.6000	1.0000	0.6000	0.7778
	Recall	0.8571	0.8571	1.0000	0.8571	1.0000	0.8571	1.0000
	F1-score	0.6667	0.8571	0.8235	0.7059	1.0000	0.7059	0.8750
	G-measure	0.6838	0.8571	0.8366	0.7171	1.0000	0.7171	0.8819

**Table 5:** Comprehensive statistics for information indices

Measures	Statistics	K-NN	SVM	SCUCC	CLCBR	ETCBR	LWCBR	RWCBR
Precision	MIN	0.5000	0.8571	0.7000	0.6000	0.7500	0.3333	0.7778
	AVG	0.6506	0.8857	0.9067	0.7633	0.8917	0.6578	0.9111
	STD	0.1490	0.0639	0.1362	0.1529	0.1087	0.1985	0.1217
	MAX	0.8571	1.0000	1.0000	1.0000	1.0000	0.8000	1.0000
Recall	MIN	0.7143	0.8571	0.2857	0.5714	0.7143	0.2857	0.7143
	AVG	0.8285	0.8857	0.6857	0.7714	0.8571	0.7428	0.9143
	STD	0.0639	0.0639	0.2748	0.1917	0.1429	0.3097	0.1278
	MAX	0.8571	1.0000	1.0000	1.0000	1.0000	1.0000	1.0000
F1-score	MIN	0.5882	0.8571	0.4444	0.6154	0.7692	0.3077	0.8333
	AVG	0.7236	0.8857	0.7375	0.7576	0.8672	0.6861	0.9013
	STD	0.1067	0.0639	0.1795	0.1514	0.0965	0.2320	0.0637
	MAX	0.8571	1.0000	0.9231	1.0000	1.0000	0.8750	1.0000
G-measure	MIN	0.5976	0.8571	0.5345	0.6171	0.7715	0.3086	0.8452
	AVG	0.7221	0.8857	0.7649	0.7624	0.8708	0.6931	0.9070
	STD	0.1088	0.0639	0.1451	0.1488	0.0951	0.2345	0.0593
	MAX	0.8571	1.0000	0.9258	1.0000	1.0000	0.8819	1.0000

Note: *MIN* the minimum, *AVG* average, *STD* standard deviation, *MAX* the maximum

**Fig. 2:**
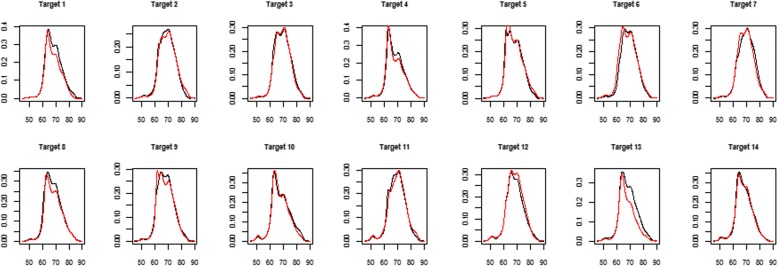
The retrieved cases by RWCBR at Fold 1. The figure illustrates 14 cases of proteomic profiles at fold 1. Black-solid lines represent target cases, and red-solid lines represent the solutions optimized by RWCBR

The retrieved cases for each target case from the test set by RWCBR model at the first fold are displayed on Fig. 2. The black solid lines represent the 14 target cases from the test set, and the red solid lines are the most similar cases retrieved from the case-bases according to the similarity measure of Eq. (4).

## Conclusion and Discussion

Plasma proteomic profiles have been regarded as a potential biomarker to diagnose certain diseases according to their specific patterns. It is challenging to precisely predict the clinical status based solely on the patterns of profiles because some profiles do not frequently follow the general patterns, which leads to large within-subject variance. The prediction based on CBR based approaches may be effective in that case. The CBR classifier predicts the clinical status of a target case by retrieving the most similar case from the case-base, so it would be advantageous in prediction because it can avoid the risk to make decision according to deviated overall means due to the outlying pattern. However, CBR classifier often shows low predictability, and some studies made efforts to enhance the predictability using weight optimization for features. There is still no golden standard to optimize or allocate the feature weights, which can be dependent upon the characteristics of the data we encounter.

The present study suggests a rank-based weighted CBR classifier (RWCBR) to predict the clinical status of plasma proteomic profiles. The rank-based weighted CBR classifier uses a weighted similarity based on rank-order information of distance metrics to retrieve the most similar case from the case-base where the feature weights are optimized from Wilcoxon’s rank sum statistics. We conducted numerical experiments to validate the performance of RWCBR. As reference methods, two machine learning techniques, *k*-NN and SVM, a statistical method, SCUCC, a classical CBR (CLCBR) and two differently weighted CBR, ETCBR and LWCBR methods were compared in terms of Precision, Recall, F-1 score and G-measure. According to the results, SVM showed the lowest standard deviation and the highest minimum value for Precision, Recall, but RWCBR outperform in average value in all information indices, and it maintained the lowest standard deviation in F-1 score and G-measure. Also, LWCBR showed lower performance than CLCBR in most information indices. A weighted CBR approaches do not always perform well, so the weight allocation or optimization methods should take into accounts the characteristics of the data set to enhance the performance of CBR classifier.

The sample size of the plasma proteomic profiles was small in the present study. However, RWCBR approach showed potential to predict the clinical status based solely on plasma proteomic profiles as a robust classifier over different sample sets in the present study.

## Abbreviations

AVG: Average
CBR: Case-based reasoning
CLCBR: Classical CBR
CV: Cross-Validation
ETCBR: Entropy-based CBR
k-NN: k-Nearest neighbor
LWCBR: Logistic regression-based Weighted CBR
MAX: The maximum
MIN: The minimum
RWCBR: Rank-based Weighted CBR
SCUCC: Statistically classified using composite coefficient
STD: Standard deviation
SVM: Support vector machine
